# A PILOT FEASIBILITY STUDY INVESTIGATING BALANCE DELIVERY TO OLDER ADULTS WITH CHRONIC CONDITIONS IN REHAB SETTINGS

**DOI:** 10.1093/geroni/igad104.3747

**Published:** 2023-12-21

**Authors:** Elizabeth Choma, Jackson Peckenpaugh, Diane Treat-Jaconson, Lauren Martin, Julian Wolfson, Manda Keller-Ross, Siobhan McMahon

**Affiliations:** Whitworth University, Spokane, Washington, United States; Eastern Washington University, Spokane, Washington, United States; University of Minnesota, Minneapolis, Minnesota, United States; University of Minnesota, Minneapolis, Minnesota, United States; University of Minnesota, Minneapolis, Minnesota, United States; University of Minnesota, Minneapolis, Minnesota, United States; University of Minnesota, Minneapolis, Minnesota, United States

## Abstract

Little is known about the integration of balance exercise delivery into physical rehabilitation settings for older individuals with chronic conditions and fall risk. We assessed the feasibility, acceptability and limited efficacy of delivering evidence-based balance exercises to older adults with fall risk enrolled in cardiac rehabilitation (CR) for peripheral artery and/or cardiovascular disease through an explanatory sequential mixed methods pilot study. Phase one included a two-arm randomized trial comparing feasibility, acceptability and functional balance among 10 participants receiving an 8-week balance program delivered adjacent to CR and 10 participants receiving CR only. Phase two included semi-structured interviews to elicit perceived barriers/facilitators of program receipt. Data was analyzed using descriptive statistics to summarize feasibility and acceptability indicators and one-way ANCOVA to test balance effects (Short Physical Performance Battery [SPPB], Mini-BESTest). Qualitative data was analyzed through content analysis. Participants had a mean age of 72.3 (7.3). On average, treatment group participants attended 7.2 (1.8) out of 8 meetings and self-reported completion of 4.5 (1.8) exercise sessions/week. Adjusting for baseline values, the treatment group exhibited greater changes in the SPPB than the usual care group after 8 weeks (10.71±0.36 vs. 9.59±0.36; adjusted difference=1.13 [95% CI, 0.3,2.2]; p=0.045). Mini-BESTest scores were not significantly different (p=0.061). Participants expressed satisfaction with program duration and frequency, quality of instructional materials, challenge level, ease of home exercise completion, convenient delivery location, and improved balance self-awareness/skills. These findings could inform the design of future efficacy trials investigating balance integration within CR settings to improve reach of fall prevention efforts.

